# Intercropping with Pigeonpea (*Cajanus cajan* L. Millsp.): An Assessment of Its Influence on the Assemblage of Pollinators and Yield of Neighbouring Non-Leguminous Crops

**DOI:** 10.3390/life13010193

**Published:** 2023-01-09

**Authors:** Ujjwal Layek, Arijit Kundu, Nandita Das, Rajib Mondal, Prakash Karmakar

**Affiliations:** 1 Department of Botany, Rampurhat College, Birbhum 731224, India; 2 Department of Botany & Forestry, Vidyasagar University, Midnapore 721102, India; 3 Centre for Life-Sciences, Vidyasagar University, Midnapore 721102, India

**Keywords:** carpenter bee, co-blooming crop, leafcutter bee, pigeonpea, pollinator

## Abstract

Intercropping is practiced in modern intensive agriculture considering many benefits, including additive crop yield. However, it may have competitive or facilitative interactions between pollinator-dependant crops. Here, we investigated the reproductive aspects of pigeonpea (*Cajanus cajan*)**.** We assessed the influence of blooming pigeonpea on pollinator’s assemblage and the yield of neighbouring non-leguminous crops (e.g., coriander, mustard). For these, we recorded floral visitors and the yield of the targeted crops from two types of fields―closely situated and distantly situated concerning pigeonpea plantation. Pigeonpea is autogamous, but pollinator’s visits enhance fruit and seed sets. Bright, nectariferous flowers emitted several volatile organic compounds and were visited by numerous insect species. The prime pollinators of pigeonpea are carpenter bees and leafcutter bees. In contrast, halictidae, honeybees and stingless bees mainly pollinate the co-blooming non-leguminous crops (coriander and mustard). The richness and abundance of pollinators on these co-blooming crops remain similar in closely situated and distantly situated fields. As a result, the yield of the neighbouring crops is not significantly influenced by the blooming pigeonpea. Therefore, it can be concluded that planting pigeonpea in ridges of agricultural fields will be an additional agricultural output without affecting the assemblage of pollinators and yields of neighbouring co-blooming crops.

## 1. Introduction

Crop yield is a highly complex attribute influenced by many variables, including genotype, environment, pollinators, and their interactions. Information on the reproductive biology of crop plants is crucial for suggesting appropriate management practices [1]. Pollination is a key component of effective plant reproduction, making pollinators an essential part of the world’s biodiversity and a source of essential ecosystem services for both cultivated and native plants [2]. More than 75% of agricultural crops and wild plant species depend upon insect pollination [3]. Pollinators are declining as a result of anthropogenic factors such as habitat loss and fragmentation, agrochemical use, pathogens and parasites, alien species invasion, and climate change [2,4]. Pollinator declines can result in the loss of pollination services, which significantly negatively impacts the maintenance of biological diversity, broader ecosystem stability, crop production, food security and human welfare. A reduction in pollinator service can directly influence reproductive output, decreasing the quantity and/or quality of fruit and seed sets [5,6]. Pollinator crisis also increases the selfing rate and thus causes an increase in inbreeding depression [7,8].

In low-input agriculture systems in tropical regions, intercropping techniques are now widely used. Intercropping in some crops can cause efficient land use, which is an important component of sustainable agriculture [9]. Furthermore, cropping of several plant species together may reduce the adverse effects of a monoculture and thus can be employed in ecological agricultural systems. We think that the ridge of agricultural fields can be utilized in intercropping systems. *Cajanus cajan* (L.) Millsp. (commonly known as pigeonpea) may be a suitable crop for that purpose. It is a multipurpose drought-tolerant crop cultivated mainly for its edible seeds, which are high in dietary protein. It is also an abundant source of minerals and vitamins [10]. The plant also has ethnomedicinal uses. The infusion of leaves is used to treat anaemia, hepatitis, diabetes, urinary infections, yellow fever, and genital and other skin irritations (especially in females). Floral decoctions are used for bronchitis, cough and cold, pneumonia, dysentery and menstrual disorders [11]. The active constituents in the leaves and seeds are alkaloids, cyanogenic glycosides, flavonoids, saponins and tannins [12]. In addition, *Cajanus cajan* is a natural barrier to soil erosion and a biological factory for fixing atmospheric nitrogen in the soil.

In sustainable agricultural practices, adequate knowledge about the reproductive ecology of the crop is essential. Additionally, data on pollinators, their activity and interactions with other co-blooming crops are also helpful in sustaining the agricultural outputs. Here, we hypothesize that planting pigeonpea in agricultural ridges as intercropping with other co-blooming non-leguminous crops may be an effective agricultural practice to enhance the additive crop yield. Pigeonpea may influence (positively or negatively) the interactions of pollinators with co-blooming crops and, thereby, affect the outcomes of such co-blooming crops. Therefore, the current work aimed to assess the floral biology and pollination ecology of pigeonpea and its influence on the assemblage of pollinators and the yield of neighbouring co-blooming crops.

## 2. Materials and Methods

### 2.1. Plant Material and Study Site

The present study was conducted on pigeonpea (*Cajanus cajan* (L.) Millsp.), belonging to the plant family Fabaceae. Additionally, we recorded floral visitors and the yield of two co-blooming crops, *Brassica juncea* (L.) Czern. (commonly known as mustard) and *Coriandrum sativum* L. (commonly known as coriander).

The study was conducted in Lalpur (22.86° N and 87.35° E) and Jenadihi (23.4468° N and 87.0449° E) in Bankura district and Chandannagar (22.5153° N and 882147° E) of Hooghly district in West Bengal, India, from 2020 to 2022. Reproductive aspects of pigeonpea were studied for 40 plants in Lalpur and for five plants in Chandannagar. For recording floral visitors of the co-blooming plants (coriander and mustard), we selected the crop fields in Lalpur and Jenadihi in two ways―closely situated (i.e., adjacent to pigeonpea patch; *n* = 3 for each crop) and distantly situated (>500 m distance from the pigeonpea patch; *n* = 4 for each crop). 

### 2.2. Floral Biology of Pigeonpea

The total number of inflorescences per individual was calculated using a random sample of 20 plants. The total number of flowers per inflorescence (*n* = 20), flowering period, flower development time, flower longevity, and anthesis duration were all recorded. To determine the flower development time, we selected very small flower buds (*n* = 10 buds in 10 inflorescences per morphotype) and marked them by putting black dots of ink on the calyx. Then, we recorded the date of opening of these buds as mature flowers by continuing field surveys. We tagged a few inflorescences to determine the longevity of an individual flower. Mature flower buds were marked with black ink dots on their calyx (*n* = 20 for each morphotype). We noted the date and time of flower opening and senescence (considering the time of closing of petals). We measured the dimensions (length and/or breadth) of floral parts (*n* = 10 for each part). 

To count the pollen grains, we collected the ten anthers from a mature flower bud (*n* = 10 flowers for each month). The pollen grains were then extracted from the anthers by adding 1 mL of 0.4 M sucrose solution and crushing with a glass rod. The solution was then filtrated to remove the debris. Following gentle shaking, 10 μL of the sample solution was pipetted onto a glass slide using a micropipette, and the number of pollen grains suspended in the solution was counted. We replicated the above procedure three times for each sample, estimating the average number of pollen grains in 10 μL and then multiplying by 100 to obtain the total number of pollen grains per flower [13]. To study pollen morphology, pollen grains were acetolysed by the Erdtman’s [14] method to increase the visibility of diagnostic characters. Leica DM1000 (Wetzlar, Germany) was used for microscopic analysis, while Leica DFC295 was used to capture microphotographs of pollen grains at the appropriate magnifications. By rupturing the ovary wall, the number of ovules was directly counted (*n* = 10 per morphotype). 

The benzidine-H_2_O_2_ test was used to assess stigma receptivity [15]. We measured receptivity every 6 h, beginning with the flower’s opening (i.e., 0 h), then every 6, 12, 18, and 24 h. To measure pollen viability (during the flower opening period and later phases, as mentioned earlier for stigma receptivity), both the staining method and the in vitro germination test were applied. A few mature flower buds were bagged with nylon net in the late afternoon. On next day, we observed them and documented their opening time. These flowers (whose opening time was known) were utilized to estimate stigma’s receptivity and pollen viability. To test receptivity, fresh stigmas (with stylar parts) were cut and immersed into a large droplet of the test solution (benzidine-H_2_O_2_) taken on a glass slide. The stigmas were inspected (the appearance of bubbles was observed) under a magnifier or a simple microscope. Regarding the staining method of pollen viability estimation, pollen grains were stained with an aqueous solution of 1% TTC, i.e., 2,3,5-triphenyl tetrazolium chloride [16]. For that, a drop of staining solution was taken on a glass slide. Pollen grains were added to this solution and wrapped in a coverslip. After 2 h of incubation, we examined the pollen grains under a light microscope. Pollen grains stained orange or bright red were considered viable. For in vitro germination test of pollen viability estimation, we used a 10% sucrose solution with 300 ppm boric acid (made of 10 gm sucrose and 30 gm boric acid dissolved in 100 mL distilled water). One drop of the solution was placed on a glass slide, pollen grains were added, and the slide was covered with a coverslip. We placed the glass slide in a petri dish with wet filter papers and incubated it for 24 h at room temperature in darkness. Then, we observed the pollen grains and counted germinated pollens with a light microscope.

For floral volatiles analysis, newly opened flowers were collected and filled (*n* = 4 flowers per vial) inside a 20 mL glass vial (Thermo Fisher Scientific, Waltham, MA, USA). The capped vials containing flowers were incubated for 3–4 h at 20–25 °C. The volatile organic compounds (VOCs) were emitted from the flowers and retained within the vials [17]. Then, vials (containing floral VOCs) were loaded into an autosampler (TriPlus RSH, Thermo Fisher Scientific, Waltham, MA, USA), and 1.5 µL of the sample was injected into a gas chromatography system (Trace 1300, Thermo Fisher Scientific, Waltham, MA, USA) using a splitless injector. We used a TG-WAX MS column (30 m × 0.25 mm × 0.25 µm) and helium as carrier gas at a flow rate of 1.20 mL/min to separate volatile compounds in the system. The temperature was adjusted to 60 °C for 5 min and then increased to 240 °C, gaining 6 °C/min and fixing the transfer line at 240 °C. To eliminate background errors, we took a blank air sample (vial with no flowers) and used it as a control for the analysis. The gas chromatograph was linked to a mass spectrometer (ISQ QD Single Quadrupole, Thermo Fisher Scientific, Waltham, MA, USA). The mass scan range was set at 40–400 a.m.u., and the scan rate was 70 eV. The volatile compounds were tentatively identified by comparing the retention indices and mass spectra to the reference standards given in the NIST 2017 collection. 

### 2.3. Breeding System of Pigeonpea

To determine the breeding system, we carried out five pollination treatments: (1) spontaneous autogamy to test for the potential of autonomous self-pollination, (2) openpollination to test the pollination success in nature provided by native pollinators, (3) manual selfing (using pollen of the same flower) to test for self-compatibility, (4) manual cross-pollination (xenogamy) to test outcrossing ability, and (5) supplementary pollination to estimate the maximum potential of reproductive outputs (fruit and seed set) of the plant species. We chose 200 matured flower buds for each morphotype for open-pollination treatment upon 20 sampling days (10 flower buds per sampling day). We used 100 flower buds for each morphotype in the other treatments. Individual flower buds were identified by placing a black dot of ink on their calyx, and inflorescences were tagged. All selected buds were covered with nylon net, with the exception of open and supplementary pollination treatments. Before anther dehiscence, selected buds were emasculated for cross-pollination treatment. We pollinated the opened flowers within 2 h of opening (at the time when the stigma attained its receptivity) to conduct the manual pollination treatments (selfing, cross, and supplementary pollination). For these manual pollination treatments, we used pollens of freshly opened (1st day) flowers. We used pollen grains from two flowers from two plants (excluding the plant bearing the emasculated flower) of the same morphotype to cross-pollinate the selected flowers. In supplementary pollination treatment, as flowers were in the open, they also received pollination services provided by native pollinators. We counted the number of fruits for each treatment and then estimated the fruit set. We calculated the index of self-incompatibility (ISI) in the same way as Raduski et al. [18]:

$$\mathrm{ISI}=1-\frac{\mathrm{Fruit}\;\mathrm{set}\;\mathrm{in}\;\mathrm{selfed}\;\mathrm{success}}{\mathrm{Fruit}\;\mathrm{set}\;\mathrm{in}\;\mathrm{outcrossed}\;\mathrm{success}}$$

Based on the ISI value, we classified the plant species into one of three categories: (i) self-incompatibility (ISI ≥ 0.8), (ii) partial self-incompatibility (0.2 < ISI < 0.8), and (iii) self-compatibility (ISI ≤ 0.2). 

To determine if plant species encounter pollen transfer constraints in nature, we calculated the “coefficient of pollination deficit (*D*)” using the method of Layek et al. [19] as follows:

$$D=1-\frac{\mathrm{Ro}}{\mathrm{Rs}\;}$$

where Ro denotes reproductive success (i.e., percentages of fruit set) in open pollination, and Rs means reproductive success in supplementary pollination. We classified plant species into four categories based on the calculated value of *D*: (a) high pollination deficit (*D* > 0.5), (b) medium pollination deficit (*D* = 0.3–0.5), (c) low pollination deficit (0.3 > *D* ≥ 0.1), and (d) negligible pollination deficit (*D* < 0.1). 

### 2.4. Floral Visitors of Pigeonpea

We observed visitors at six time-slots: (i) 6.00–8.00 h, (ii) 8.00–10.00 h, (iii) 10.00–12.00 h, (iv) 12.00–14.00 h, (v) 14.00–16.00 h, and (vi) 16.00–18.00 h, during peak flowering (i.e., mid-November to mid-January) of the two flowering sessions (i.e., September 2020–February 2021 and September 2021–February 2022). A direct observation method was used to encounter floral visitors [13]. To count the number of visiting insects, one survey (i.e., plant-based sampling) period lasts 5 min and covers approximately 15–30 flowers. We completed 240 surveys (20 surveys per time slot/session). Visitors were identified (up to species level) in the field or captured (using an insect net) for later identification. The captured insects were placed in a glass vial with a piece of cotton (soaked in ethyl acetate) and later identified by entomologists (at the ZSI, Kolkata). According to Layek et al. [19], the relative abundance (RA) of each flower-visiting species was determined as follows:

$$\mathrm{RA}\;\left(\%\right)=\frac{ni}{N}\times 100$$

where n*i* is the number of encounters with the insect species *i*, and N is the total number of encounters with all flower visitors on the plant species. 

The number of flowers visited in 1 min duration was used to estimate the flower visitation rate of visitors. We collected data (number of flowers visited per minute) for an insect species 30 times. However, for the insect species that spent much time on flowers (e.g., butterflies and stingless bees), the recording time was extended to 10 min, and the obtained value (i.e., the number of flowers visited) was divided by 10 to convert the value into a 1 min duration.

To estimate the number of pollen grains carried by a visitor species, we entrapped the visitors (*n* ≥ 5 for each dominant species; we excluded insect species with sample sizes less than 5) with an insect net. We noticed whether stacked pollen loads were present or not by closely inspecting the captured bee species with a 10× hand lens. In the case of carpenter bees, honeybees, leafcutter bees, and solitary bees, we looked at body surface pollen grains (which exclude stacked pollen loads on corbiculae, scopae, or other parts) and stacked pollen loads in different ways. The hind legs of visitors were amputated and placed in a glass vial separately to count the stacked pollen grains. To count stacked pollen grains on the abdomen (in the case of leafcutter bees), we first estimated the total body surface pollen grain content of pollen (or mixed) foragers and then calculated the average number of body surface pollen grains of a nectar forager (i.e., the forager which was without stack pollen load). To estimate the content of body surface pollen grains, we added 1.0–5.0 mL (relying on the body size of the captured visitor) of 0.4 M sucrose solution to the vials containing the bodies of insects. We vigorously shook them to wash out the pollen grains. The pollen grains were then counted using the method mentioned earlier (for the pollen count of flowers). We only looked at the pollen grains of the plants we were interested in and ignored any other pollen types that were present. 

According to Layek et al. [13], we calculated an ‘approximate pollination value’ (APV) for the flower-visiting species in order to estimate the significance of the floral visitors as pollinators of the plant species as follows:

APV=RA×VR×PCV

In this case, RA stands for the relative abundance of a flower-visiting species, VR for the visitation rate, and PCV for the pollen-carrying value of the insect species. The pollen-carrying value (PCV) of floral visitors is estimated according to the method of Layek et al. [13]. It is derived from the summation of two components: (i) PCV 1 (derived from body surface pollen content omitting stack pollen loads on abdomen, corbiculae or scopae; ranging from 0 to 5) and (ii) PCV 2 (calculated from stack pollen content on abdomen, corbiculae or scopae; ranging from 0 to 3) (Supplementary Table S1).

### 2.5. Influence of Blooming Pigeonpea on the Pollinator’s Assemblage of Co-Blooming Crops

We observed the visitors of co-blooming crops (coriander and mustard) at different time-slots similar to the time-slots utilized for pigeonpea from mid-November to mid-January. To measure the abundance of the floral visitors, we recorded the number of encountered individuals of different insect species in 1 m^2^ field area per 5 min over the six time-slots. The abundance of visitors was recorded separately for closely and distantly situated fields. The relative abundance (RA) of visitors was also calculated. After estimating the pollen-carrying value of some dominant visitors, an approximate pollination value (APV) was also determined.

### 2.6. Influence of Blooming Pigeonpea on the Yield of Co-Blooming Crops

For estimation of the yield of co-blooming crops, we selected an area of 5 m × 5 m for harvesting the ripened fruits. We took the fruit and seed weights for coriander and mustard, respectively. The obtained values were multiplied by 400 to convert the yield for the hectare area. The obtained yield in the closely situated fields was compared with the distantly situated fields to estimate the impact of blooming pigeonpea on the yield of neighbouring co-blooming crops.

### 2.7. Statistical Analyses

The mean and standard deviation were calculated using descriptive data analysis. We used the Shapiro–Wilk tests to determine whether the data were normally distributed. We used a parametric test, one-way ANOVA, on normally distributed data, and *p* ≤ 0.05 was deemed statistically significant. If the obtained *p* value was significant, we conducted DMRT (as a post hoc test) to evaluate the significant difference among the mean values. To judge whether the abundance of pollinators and crop yield varied between the distantly situated and closely situated fields, we performed an independent *t* test. The statistical analyses mentioned above were carried out using SPSS (16.0) and Microsoft Excel. 

## 3. Results

### 3.1. Floral Biology of Pigeonpea

**Figure 1:**
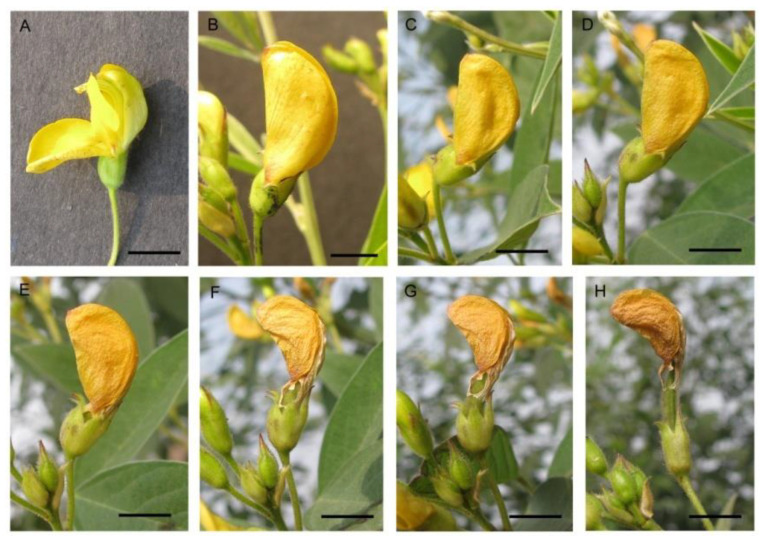
Flowers at different times after opening: (**A**) 0 h, (**B**) 24 h, (**C**) 48 h, (**D**) 72 h, (**E**) 96 h, (**F**) 120 h, (**G**) 144 h, and (**H**) 168 h. Scale bar = 5 mm.

**Figure 2:**
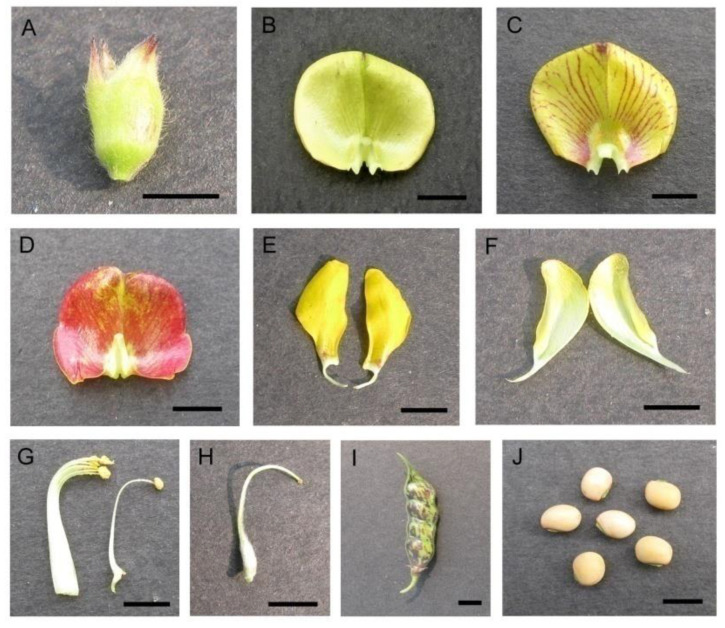
(**A**) Calyx, (**B**–**D**) vexillum, (**E**) wings, (**F**) keels, (**G**) androecium, (**H**) gynoecium, (**I**) fruit, and (**J**) seeds. Scale bar = 5 mm.

**Figure 3:**
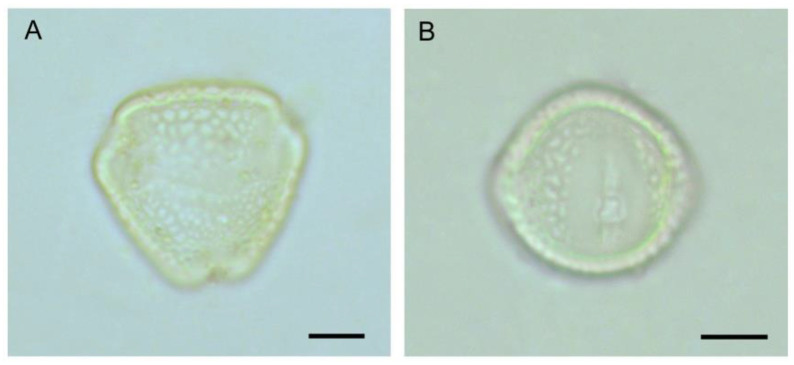
Pollen grain of *Cajanus cajan*: (**A**) polar view, (**B**) equatorial view. Scale bars = 10 µm.

The flowering of pigeonpea occurred from September to February, with the peak in mid-November to mid-January. Flowers opened throughout the daytime, peaking at 8.00–10.00 h. Flowers’ longevity (in the open state) was about 6–10 h. The senescence of floral parts takes place slowly (Figure 1). In most cultivars, flowers are borne on the terminal or auxiliary racemes (4–12 cm) and are carried on a long peduncle. Pedicels are about 11.10 ± 2.02 mm long. Flowers are zygomorphic, papilionaceous, calyx campanulate, pubescent with five sepals, about 7.00 ± 0.67 mm long, with a green lower part, and the sepals tips are reddish. The vexillary petal (16.85 mm × 16.95 mm) is basally inflexed, biauriculate. Based on colour-ornamentation, we recognised three morpho-types (Figure 2): (i) morph 1—yellow coloured without veins, (ii) morph 2—yellow coloured with reddish veins, and (iii) morph 3—red coloured. The wings are obliquely obovate with an incurved claw. The keel petals are obtuse (round), incurved and boat-shaped. The keel covers the androecium (male organs) and gynoecium (female organs) of the flower. Stamens diadelphus (9 + 1), filaments are 16.0 ± 1.08 mm in length, tapering towards the top, greenish-white in colour; anthers ellipsoid, dorsifixed, yellowish, and slit longitudinal. About 30,033.33 ± 2599.88 pollen grains are produced per flower. Pollen grains are 3-colporate, prolate-spheroidal (32 µm × 30 µm), amb triangular, and reticulate exine ornamentation (Figure 3). The ovary is elongated, about 6.1 ± 0.64 mm long, greenish, deeply haired, 1 chambered; style 13.2 ± 0.95 mm long, greenish; stigma simple. Pods are linear-oblong, compressed, bi-valved, and depressed between the seeds. The seed set per pod significantly differed among the three morpho-types (F_2,247_ = 11.23, *p* < 0.001). Morph-3 (red vexillum) has higher seeds per fruit (4.18 ± 0.80 seeds/fruit) than morph-1 (3.74 ± 0.77 seeds/fruit) and morph-2 (3.57 ± 0.69 seeds/fruit). Seeds are sub-spherical, about 6 mm × 4 mm × 1.5 mm, and seed coat colour varies from light brown to reddish.

From the GC-MS analysis of floral volatile organic compounds (VOCs), we identified 26 compounds (Table 1). Among them, retention time was lower in Pentanal, Hexanal, Heptanal, 1-pentanol, cis-Hept-2-enal; and higher in 2-Oxo-2-phenylethyl formate, (2E)-2-[(4-Nitrophenyl) imino]-1-phenylethanone, 1-Hexyl-2-nitrocyclohexane, 1-Methylene-2b-hydroxymethyl-3,3-dimethyl-4b-(3-methylbut-2-enyl)-cyclohexane, Methyl (2E)-2-methoxy-2-butenoate, n-Propionylurea, and Diethyl phthalate. Relative abundance was higher in Diethyl phthalate, 4-Methyl-3-heptanone, 2,4-Decadienal, 2-Oxo-2-phenylethyl formate, 1-Hexyl-2-nitrocyclohexane, and Caproic acid.

Anther dehiscence starts simultaneously with flower opening. At the time of anther dehiscence, viability was highest (81.24 ± 5.63% pollen stained and 73.98 ± 5.55% germinated) and then gradually decreased (Table 2). At the time of flower opening, bubbles came out from the most (85 ± 10.80%) stigmatic surface during the benzidine-H_2_O_2_ test. The proportion of receptive stigma was reduced over time. After 24 h of opening (i.e., on the next day), only 41 ± 11.97% of stigma showed receptivity.

### 3.2. Mating Systems of Pigeonpea

Fruit sets take place in all five pollination treatments: autonomous selfing, open-pollination, manual selfing, manual crossing, and supplementary pollination (Table 3). However, the percentages of the fruit set differed significantly (F_4,175_ = 37.81, *p* < 0.001). The highest fruit set was obtained in supplementary pollination treatment (63.33 ± 12.69%) and the lowest in bagged flowers, i.e., via autonomous selfing (27.67 ± 8.98%). Fruit set in open condition was comparatively lower than the manual pollination treatments. The index of self-incompatibility (ISI) was very low (0.04), suggesting that the plant is fully self-compatible. According to the coefficient of pollination deficit (*D* = 0.19), plant species showed a low deficit of pollen transfer in open conditions. Considering the different morphotypes, fruit sets also differed in various treatments. In open-pollination, morph-2 (with distinct veins in the vexillum) has a greater fruit set (57.50 ± 16.50%) than morph-1 and morph-3.

### 3.3. Floral Visitors of Pigeonpea

**Figure 4:**
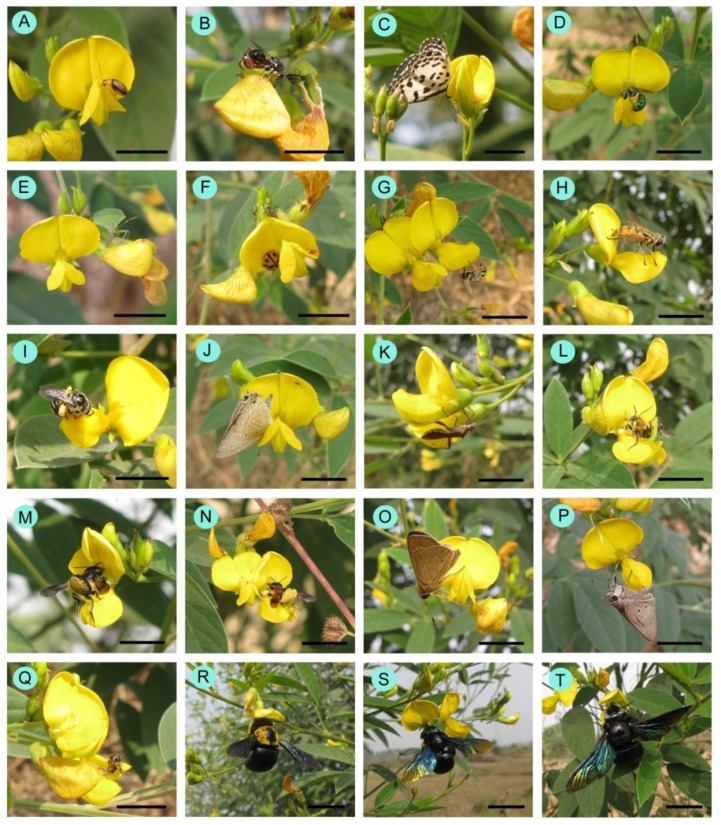
Floral visitors of *Cajanus cajan*: (**A**) *Aulacophora cinta*, (**B**) *Brachymeria* sp., (**C**) *Castalinus rosimon*, (**D**) *Ceratina binghami*, (**E**) *Chinavia hilaris*, (**F**) *Coccinella sexmaculata*, (**G**) *Episyrphus balteatus*, (**H**) *Eristalinus megacephalus*, (**I**) *Halictus acrocephalus*, (**J**) *Lampides boeticus*, (**K**) *Leptocorisa acuta*, (**L**) *Megachile conjuncta*, (**M**) *Megachile disjuncta*, (**N**) *Megachile lanata*, (**O**) *Pelopidus mathias*, (**P**) *Suastus gremius*, (**Q**) *Tetragonula iridipennis*, (**R**) *Xylocopa aestuans*, (**S**) *Xylocopa fenestrata*, (**T**) *Xylocopa latipes*. Scale bar = 5 mm.

**Figure 5:**
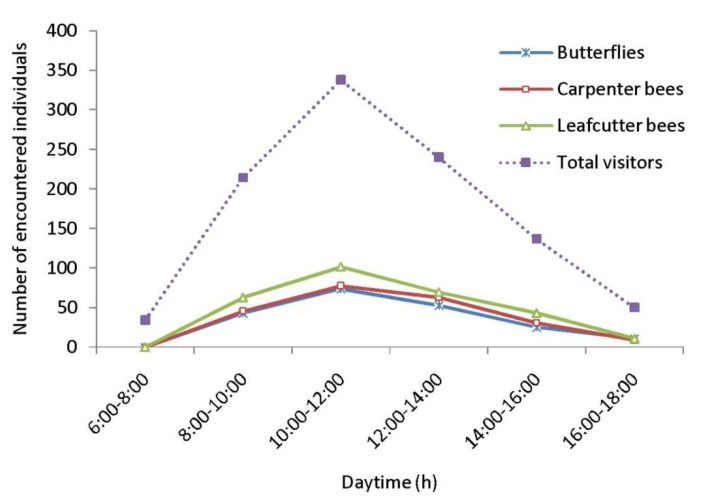
Daytime-wise abundance of floral visitors.

As floral visitors of *Cajanus cajan*, 33 insect species were identified (Table 4, Figure 4). Within the visitor’s spectrum, the order Hymenoptera is represented by 19 species, followed by Lepidoptera (6 species), Coleoptera (3 species), Diptera (3 species), and Hemiptera (2 species). The most encountered insect groups are leafcutter bees, carpenter bees and butterflies. The most abundant flower-visiting species are *Megachile disjuncta* (RA = 15.20%), *Megachile lanata* (RA = 11.55%), *Xylocopa fenestrata* (RA = 10.27%), *Xylocopa aestuans* (RA = 9.38%), *Suastus gremius* (RA = 7.01%), and *Pelopidus mathias* (RA = 5.73%). According to hours of the day, the numbers of encountered floral visitors were higher during 10.00–12.00 h (Figure 5) and lower in the early morning (6.00–8.00 h) and late afternoon (16.00–18.00 h). 

Carpenter bees, flies, halictidae, honeybees, leafcutter bees, and stingless bees collected both nectar and pollen from the flowers. Other insect groups such as ants, beetles, bugs, butterflies, and wasps collected only nectar as floral resources. Some parasitic wasps (*Brachymeria* sp.) visited the flowers for their own reproduction purposes.

The flower visitation rate significantly varied from species to species (*F*_41,958_ = 31.64, *p* < 0.001). The visitation rate was higher in Hymenopteran members (especially carpenter bees, honeybees, and leafcutter bees) than in Dipteran and Lepidopteran members (Table 4). The amount of time spent per flower also differed. Butterflies and stingless bees spent the most amount of time and carpenter bees spent the least amount of time on a flower within a single visit. It is notable that leafcutter bees attempted to visit both opened flowers and matured un-open flowers. In most cases, they opened the previously un-opened flower during their visits. Their visitation rate is also influenced by the two states of flowers. When they visited matured un-opened flowers, they spent more time (*Megachile conjuncta*: 9.92 ± 1.73 sec/visit; *Megachile disjuncta*: 14.22 ± 3.24 sec/visit; *Megachile lanata*: 11.52 ± 2.18 sec/visit) than with the opened flowers (*Megachile conjuncta*: 4.28 ± 1.98 sec/visit; *Megachile disjuncta*: 5.04 ± 2.79 sec/visit; *Megachile lanata*: 4.63 ± 2.44 sec/visit). 

Pollen-carrying values differed among the insect groups. Members of Apidae and Megachilidae carried more pollen compared to other insect families (Supplementary Table S2). The combined parameter ‘approximate pollination value (APV)’ was higher in *Megachile disjuncta*, *Megachile lanata*, *Xylocopa aestuans*, and *Xylocopa fenestrata* (Table 4). In insect group-wise consideration, APV was much higher in carpenter bees and leafcutter bees than in butterflies, flies, honeybees, other solitary bees, and wasps. In this regard, we can say that carpenter and leafcutter bees mainly pollinate the pigeonpea flowers. 

### 3.4. Influence of Blooming Pigeonpea on Pollinator Assemblage of Neighbouring Crops

In distantly situated fields (i.e., in the absence of the influence of blooming pigeonpea), we recorded 44 flower-visiting species for *Brassica juncea* (Supplementary Table S3) and 26 insect species for *Coriandrum sativum* (Supplementary Table S4). In closely situated fields (i.e., in the presence of the influence of blooming pigeonpea), species richness remained almost the same. In closely situated fields, we recorded leafcutter bees as floral visitors for *Brassica juncea*. The abundance of floral visitors does not significantly differ between the distantly situated and closely situated fields of *Brassica juncea* (df =118, *p* = 0.23; 9.32 ± 5.02 visitors/m^2^ /5 min in distantly situated fields and 10.48 ± 5.55 visitors/m^2^ /5 min in distantly situated fields) and *Coriendrum sativum* (df = 118, *p* = 0.59; 4.70 ± 2.58 visitors/m^2^ /5 min in distantly situated fields and 4.95 ± 2.59 visitors/m^2^ /5 min in distantly situated fields). The neighbouring co-blooming crops, coriander, shared some common visitors (e.g., *Apis* spp., *Episyrphus balteatus*, *Halictus acrocephalus*, and *Tetragonula iridipennis*) with pigeonpea, and mustard shared some common visitors (e.g., *Apis* spp., *Episyrphus balteatus*, *Halictus acrocephalus*, *Lasioglossum funebre*, *Megachile* spp., *Tetragonula iridipennis*, and *Xylocopa* spp.) with pigeonpea. Important pollinators of these co-blooming crops (coriander and mustard) were *Apis cerana*, *Apis florea*, *Halictus acrocephalus*, and *Tetragonula iridipennis*.

### 3.5. Influence of Blooming Pigeonpea on Yield of Neighbouring Crops 

The yield of selected crops did not significantly vary (*Brassica juncea*: df = 6, *p* = 0.53; *Coriandrum sativum*: df = 6, *p* = 0.63) between the two field types (i.e., distantly and closely situated fields). Although, the yields were slightly (non-significant at 5% level) higher in the closely situated fields (*Brassica juncea*: 1080 ± 56.10 kg/ha; *Coriandrum sativum*: 667 ± 57.63 kg/ha) than in the distantly situated fields (Table 5).

## 4. Discussion

Pigeonpea flowering occurred in West Bengal from November to February, a similar time reported in other Indian states [20]. During this time span, other entomophilous blooming crops were coriander and mustard. Flowers of pigeonpea open throughout the daytime, peaking at 8.00–10.00 h. Individual flowers lasted for about 6–10 h. However, a longer life span (>2 days) was also reported by Kar and Dutta [20]. We found three morphotypes of pigeon pea flowers. Flowers provide visitors with nectar, pollen, and floral tissues and emit several volatile compounds. Some are common to other plant species such as *Chromolaena odorata* [13] and *Foeniculum vulgare* [21]. A large number of insect species visited pigeonpea flowers. Species composition differed from the visitor’s spectrum of the plant species reported from other Indian states [22]. The diversity and abundance of visitors largely depend on geographical location and surrounding vegetation [23,24]. The dominant and effective pollinators of pigeonpea in West Bengal are carpenter bees and leafcutter bees.

Managing ecosystem services, such as pollination, that maximise crop yields are currently one of the most severe difficulties in sustainable agricultural production. Most initiatives to boost the services provided by wild pollinators focus on the management of habitats inside farms or natural habitats around farms. Co-blooming plants create resource pulses that may be important determinants of pollinator dynamics. However, it needs to be clarified how this affects the pollination and yields of other co-blooming crops. In some cases, co-blooming plants increase the abundance of pollinators and yield of the surrounding crops [24,25]. In contrast, some workers also find that co-blooming plants may reduce the reproductive output of neighbouring plants by transient pollinator dilution [26]. Our current study revealed that blooming papilionaceous pigeonpea has no significant influence on pollinator assemblage and yield of non-papilionaceous adjacent crops (coriander and mustard). This may be due to the dominant visitors of pigeonpea and non-leguminous co-blooming crops being different. The selected non-leguminous co-blooming crops are mainly visited by honeybees, halictidae (*Halictus acrocephalus*), and stingless bees (*Tetragonula iridipennis*), although pigeonpea and the other two co-blooming crops shared some common pollinators. However, the pollination efficiency of the visitors varied among the studied plants. Halictidae, honeybees and stingless bees provided significant pollination services to the non-leguminous co-blooming crops. The pollination efficiency of floral visitors depends on their foraging behaviour, flowering phenology, and floral characteristics [27]. As the abundance of potent pollinators of the non-leguminous co-blooming crops did not alter, the yield of the neighbouring crops did not significantly change by the blooming pigeonpea.

## 5. Conclusions

Pigeonpea (*Cajanus cajan*) is self-compatible and partially dependent on insect pollinators for reproductive success. Diverse insect species visit the flowers of the cover plant, while carpenter bees and leafcutter bees mainly pollinate the crop. The blooming pigeonpea plants did not significantly influence the assemblage of pollinators on neighbouring crop fields. The prime pollinators of pigeonpea (e.g., carpenter bees and leafcutter bees) do not play a pivotal role in mustard and coriander pollination. Still, other visitors such as helictidae, honeybees, and stingless bees provided important pollination services to the associated non-leguminous crops (coriander and mustard). Therefore, planting pigeonpea on the ridge of agricultural fields will be beneficial in terms of simultaneous yields, and it is without negative influence on pollinator assemblage of the co-blooming neighbouring non-leguminous crops.

## Figures and Tables

**Table 1:** Volatile organic compounds (VOCs) emitted by flowers of *Cajanus cajan*.

VOCs	Empirical Formula	Molecular Weight	Retention Time (min)
Pentanal	C_5_H_10_O	86	4.30
Hexanal	C_6_H_12_O	100	6.19
Heptanal	C_7_H_14_O	114	8.37
1-pentanol	C_5_H_12_O	88	9.86
cis-Hept-2-enal	C_7_H_12_O	112	11.54
Nonanal	C_9_H_18_O	142	13.10
(E)-2-Octen-1-al	C_8_H_14_O	126	13.87
4-Bromo-1-cyclohexene	C_6_H_9_Br	160	15.27
Pentyl glycolate	C_7_H_14_O_3_	146	17.18
trans-2-Decenal	C_10_H_18_O	154	18.27
2,4-Decadienal	C_10_H_16_O	152	21.42
Caproic acid	C_6_H_12_O_2_	116	22.01
Benzenemethanol	C_6_H_8_O	108	22.56
4-methoxy-4-methyl-1,2-pentadiene	C_7_H_12_O	112	23.87
Thujaketone	C_9_H_16_O	140	24.35
2-Nonadecanone	C_19_H_38_O	282	24.96
(z)-3-Phenylacrylaldehyde	C_9_H_8_O	182	25.35
n-Caprylic acid	C_8_H_16_O_2_	144	25.64
4-Methyl-3-heptanone	C_8_H_16_O	128	28.86
Diethyl phthalate	C_12_H_14_O_4_	222	30.39
n-Propionylurea	C_4_H_8_N_2_O_2_	116	30.99
Methyl (2E)-2-methoxy-2-butenoate	C_6_H_10_O_3_	130	31.89
1-Methylene-2b-hydroxymethyl-3,3-dimethyl-4b-(3-methylbut-2-enyl)-cyclohexane	C_15_H_26_O	222	32.04
1-Hexyl-2-nitrocyclohexane	C_12_H_23_NO_2_	213	32.71
(2E)-2-[(4-Nitrophenyl) imino]-1-phenylethanone	C_14_H_10_N_2_O_3_	254	33.47
2-Oxo-2-phenylethyl formate	C_9_H_8_O_3_	164	33.86

**Table 2:** Pollen viability and stigma receptivity of *Cajanus cajan*.

Parameters	Time Hour after Open of Flower				
	0 h	6 h	12 h	18 h	24 h
Pollen viability					
Staining with TTC (%; *n* = 10 flowers/time-slots)	81.24 ± 5.63	73.52 ± 5.46	67.87 ± 5.76	58.43 ± 5.74	50.09 ± 6.53
In vitro germination (%; *n* = 10 flowers/time-slots)	73.98 ± 5.55	68.70 ± 5.25	60.65 ± 5.25	54.84 ± 4.95	47.05 ± 4.05
Stigma receptivity					
Benzidine-H_2_O_2_ test (%; *n* = 100 stigma/time-slot)	85 ± 10.80	74 ± 10.75	64 ± 9.66	53 ± 9.49	41 ± 11.97

**Table 3:** The effects of different pollination treatments on fruit sets of *Cajanus cajan*.

Treatment	Fruit Set (%)			
	Morph-1	Morph-2	Morph-3	Average
Spontaneous autogamy (*n* = 100 per morphotype)	28 ± 7.89	31 ± 9.94	24 ± 8.43	27.67 ^d^ ± 8.98
Openpollination (*n* = 200 per morphotype)	52 ± 13.61	57.50 ± 16.50	45 ± 8.89	51.50 ^c^ ± 14.12
Manual geitonogamy (*n* = 100 per morphotype)	58 ± 14.76	59 ± 11.97	55 ± 10.80	57.33 ^bc^ ± 12.30
Manual cross-pollination (*n* = 100 per morphotype)	60 ± 14.91	61 ± 11.97	58 ± 13.17	59.67 ^ab^ ± 12.99
Supplementary pollination (*n* = 100 per morphotype)	64 ± 13.50	66 ± 14.30	60 ± 10.54	63.33 ^a^ ± 12.69

Values are given as mean ± standard deviation; means in the column followed by the same letters do not differ significantly by Duncan’s multiple range test (DMRT) at 5%.

**Table 4:** Floral visitors of *Cajanus cajan* in West Bengal.

Visitors	Relative Abundance	Floral Resources	Visitation Rate	Time Spent/Flower	APV
Coleoptera					
*Aulacophora cincta*	0.89	fl	-	-	-
*Coccinella sexmaculata*	1.28	fl	-	-	-
*Curculio* sp.	1.18	fl	-	-	-
Diptera					
*Episyrphus balteatus*	3.16	p	2.30	16.55 ± 3.80	3.63
*Eristalinus megacephalus*	2.67	p	1.80	19.83 ± 5.23	2.40
*Stomorhina* sp.	0.79	p	-	-	-
Hemiptera					
*Chinavia hilaris*	0.69	n	-	-	-
*Leptocorisa acuta*	0.49	n	-	-	-
Hymenoptera					
*Allorynchium metalicum*	1.88	n	2.93	3.78 ± 1.01	-
*Amegilla zonata*	0.89	n, p	6.60	2.45 ± 0.51	14.68
*Apis cerana*	0.69	n, p	3.77	4.20 ± 0.95	6.50
*Apis dorsata*	0.89	n, p	4.23	4.02 ± 1.04	9.41
*Apis florea*	0.49	n, p	3.60	4.57 ± 1.09	4.41
Braconid wasp	1.58	-	-	-	-
*Brachymeria* sp.	2.57	-	1.87	-	-
*Camponotus compressus*	1.09	n	-	-	-
*Ceratina binghami*	2.27	n, p	2.40	11.62 ± 2.72	10.90
*Halictus acrocephalus*	1.78	n, p	2.97	9.87 ± 2.11	13.22
*Ichneumon* sp.	1.18	n	-	-	-
*Lasioglossum funebre*	0.39	n, p	2.83	9.92 ± 2.20	-
*Megachile conjuncta*	1.78	n, p	3.13	6.16 ± 3.28	11.14
*Megachile disjuncta*	15.20	n, p	4.03	8.10 ± 5.25	214.40
*Megachile lanata*	11.55	n, p	4.47	6.93 ± 4.02	180.70
*Tetragonula iridipennis*	1.88	n, p	0.50	-	1.41
*Xylocopa aestuans*	9.38	n, p	6.10	3.36 ± 1.25	171.65
*Xylocopa fenestrata*	10.27	n, p	5.97	3.60 ± 1.32	183.94
*Xylocopa latipes*	2.76	n, p	6.33	2.97 ± 1.52	43.68
Lepidoptera					
*Castalinus rosimon*	1.68	n	0.60	-	-
*Catochrysops strato*	2.57	n	0.44	-	-
*Jamides bochus*	1.83	n	0.38	-	-
*Pelopidus mathias*	5.73	n	0.63	80.71 ± 23.43	1.80
*Suastus gremius*	7.01	n	0.65	72.75 ± 22.72	2.27
*Telicota colon*	1.48	n	0.55	-	-

note: fl—floral tissue, n—nectar, p—pollen.

**Table 5:** Impact of blooming pigeonpea on yield of neighbouring co-blooming crops.

Parameters	Yield	
	Distantly Situated Fields	Closely Situated Fields
On *Brassica juncea*		
Seed yield (kg/hectare area)	1061 ± 50.32	1080 ± 56.10
On *Coriendrum sativum*		
Fruit yield (kg/hectare area)	640 ± 57.78	667 ± 57.63

## Data Availability

Data generated or analysed during this study are fully provided within the published article and its supplementary materials.

## Supplementary Materials

The following supporting information can be downloaded at: https://www.mdpi.com/article/10.3390/life13010193/s1, Table S1: Pollen carrying value (PCV = PCV 1 + PCV 2) of floral visitors; Table S2: Pollen carrying value (PCV = PCV 1 + PCV 2) of floral visitors of Cajanus cajan; Table S3: Floral visitors of Brassica juncea in West Bengal; Table S4: Floral visitors of Coriandrum sativum in West Bengal.
