# Supplementary material for: Intercropping with Pigeonpea (Cajanus cajan L. Millsp.): An Assessment of Its Influence on the Assemblage of Pollinators and Yield of Neighbouring Non-Leguminous Crops
Source: Life (Basel). 2023 Jan 9;13(1):193. doi: 10.3390/life13010193 (PMC9866136; doi:10.3390/life13010193)
Supplement: Supplementary file 1 [file life-13-00193-s001.zip › Supplementary Table S1.pdf]

Supplementary Table S1. Pollen carrying value (PCV = PCV 1 + PCV 2) of floral visitors.

Body surface pollen content (excluding stack pollen loads)	Value of PCV 1
0	0
<100	0.5
100–200	1
>200–500	1.5
>500–1000	2
>1000–2000	2.5
>2000–5000	3
>5000–10000	3.5
>10000–20000	4
>20000–50000	4.5
>50000	5
**Stacked pollen loads on corbiculae, scopae, or abdomen**	**Value of PCV 2**
0	0
<1000	0.5
1000-5000	1
>5000-10000	1.5
>10000-20000	2
>20000-50000	2.5
>50000	3
