# Supplementary material for: Intercropping with Pigeonpea (Cajanus cajan L. Millsp.): An Assessment of Its Influence on the Assemblage of Pollinators and Yield of Neighbouring Non-Leguminous Crops
Source: Life (Basel). 2023 Jan 9;13(1):193. doi: 10.3390/life13010193 (PMC9866136; doi:10.3390/life13010193)
Supplement: Supplementary file 1 [file life-13-00193-s001.zip › Supplementary Table S2.pdf]

Supplementary Table S2. Pollen carrying value (PCV = PCV 1 + PCV 2) of floral visitors of *Cajanus cajan*.

Visitors	Body surface pollen	PCV 1	Stacked pollen	PCV 2	PCV
Coleoptera					
Aulacophora cincta	-	-	-	-	-
Coccinella sexmaculata	-	-	-	-	-
Curculio sp.	-	-	-	-	-
Diptera					
Episyrphus balteatus	86.67 ± 63.25	0.5	0	0	0.5
Eristalinus megacephalus	90 ± 62.95	0.5	0	0	0.5
Stomorhina sp.	-	-	-		
Hemiptera					
Chinavia hilaris	-	-	-		
Leptocorisa acuta	-	-	-		
Hymenoptera					
Allorynchium metalicum	-	-	-		
Amegilla zonata	360 ± 151.37	1.5	2786.67 ± 2556.97	1	2.5
Apis cerana	253.33 ± 78.88	1.5	3146.67 ± 6700.42	1	2.5
Apis dorsata	373.33 ± 148.07	1.5	2880 ± 9107.36	1	2.5
Apis florea	236.67 ± 85.27	1.5	1420 ± 4490.43	1	2.5
Braconid wasp	-	-	-		
Brachymeria sp.	-	-	-		
Camponotus compressus	-	-	-		
Ceratina binghami	180 ± 80.43	1	1676.67 ± 1543.21	1	2
Halictus acrocephalus	203.33 ± 74.45	1.5	2206.67 ± 2095.66	1	2.5
Ichneumon sp.	-	-	-	-	
Lasioglossum funebre	-	-	-	-	
Megachile conjuncta	426.67 ± 148.07	1.5	793.33 ± 1179.43	0.5	2.0
Megachile disjuncta	920 ± 210.35	2	5261.33 ± 3996.16	1.5	3.5
Megachile lanata	873.33 ± 218.75	2	5028 ± 5066.90	1.5	3.5
Tetragonula iridipennis	136.67 ± 86.71	1	803.33 ± 837.61	0.5	1.5
Xylocopa aestuans	800 ± 414.25	2	1248.15 ± 1853.83	1	3.0
Xylocopa fenestrata	816.67 ± 396.36	2	1062.96 ± 1124.90	1	3.0
Xylocopa latipes	883.33 ± 368.93	2	513.33 ± 678.63	0.5	2.5
Lepidoptera					
Castalinus rosimon	-	-	-	-	-
Catochrysops strato	-	-	-	-	-
Jamides bochus	-	-	-	-	-
Pelopidus mathias	75 ± 147.84	0.5	0	0	0.5
Suastus gremius	91.67 ± 157.42	0.5	0	0	0.5
Telicota colon	-	-	-	-	-
