# Supplementary material for: Intercropping with Pigeonpea (Cajanus cajan L. Millsp.): An Assessment of Its Influence on the Assemblage of Pollinators and Yield of Neighbouring Non-Leguminous Crops
Source: Life (Basel). 2023 Jan 9;13(1):193. doi: 10.3390/life13010193 (PMC9866136; doi:10.3390/life13010193)
Supplement: Supplementary file 1 [file life-13-00193-s001.zip › Supplementary Table S3.pdf]

Supplementary Table S3. Floral visitors of *Brassica juncea* in West Bengal.

Visitors	Abundance		Relative abundance	Floral resources	Flower visitation rate	APV
	Distantly fields	Closely fields				
Coleoptera						
Aulacophora cincta	0.03	0.03	0.30	fl	-	-
Aulacophora frontalis	0.02	0.02	0.20	fl	-	-
Cheilomenes sexmaculata	0.03	0.02	0.25	fl	-	-
Coccinella septumpunctata	0.07	0.06	0.66	fl	-	-
Monolepta signata	0.02	-	0.10	fl	-	-
Raphidopalpa foveicollis	0.06	0.05	0.56	fl	-	-
Diptera						
Episyrphus balteatus	0.13	0.15	1.41	n, p	-	-
Eristalinus megacephalus	0.1	0.11	1.06	n, p	-	-
Paragus serratus	0.09	0.09	0.91	p	-	-
Pelatachina tibialis	0.03	0.02	0.25	p	-	-
Stomorhina sp.	0.06	0.07	0.66	n, p	-	-
Syritta pipiens	0.09	0.08	0.86	n, p	-	-
Hemiptera					-	-
Agonoscelis nubila	0.04	0.05	0.45	n	-	-
Chinavia hilaris	0.06	0.06	0.61	n	-	-
Hymenoptera						
Allorynchium metalicum	0.18	0.20	1.92	n	-	-
Amegilla zonata	0.24	0.26	2.53	n, p	-	-
Antepipona ovalis	0.17	0.16	1.67	n	-	-
Apis cerana	1.08	1.26	11.82	n, p	6.80 ± 2.17	361.69
Apis dorsata	0.53	0.58	5.61	n, p	7.30 ± 2.08	163.81
Apis florea	0.91	1.03	9.80	n, p	5.95 ± 1.96	262.40
Braconid wasp	0.09	0.08	0.86	-	-	-
Brachymeria sp.	0.05	0.04	0.45	-	-	-
Camponotus compressus	0.07	0.07	0.71	n	-	-
Ceratina binghami	0.41	0.43	4.24	n, p	5.35 ± 1.84	68.05
Chalybion bengalense	0.12	0.13	1.26	n	-	-
Eumenes fraternus	0.16	0.15	1.57	n	-	-
Halictus acrocephalus	0.85	1.14	10.05	n, p	5.60 ± 1.64	196.98
Ichneumon sp.	0.13	0.14	1.36	n	-	-
Lasioglossum funebre	0.23	0.24	2.17	n, p	-	-
Megachile disjuncta	-	0.06	0.30	n, p	-	-
Megachile lanata	-	0.04	0.20	n, p	-	-
Sphecodes gibbus	0.34	0.33	3.38	n	6.20 ± 1.70	41.91
Tetragonula iridipennis	1.82	2.08	19.70	n, p	2.85 ± 0.75	140.36
Thyreus nitidulus	0.15	0.14	1.46	n	-	-
Xylocopa aestuans	0.18	0.20	1.92	n, p	4.95 ± 1.90	33.26
Xylocopa fenestrata	0.21	0.24	2.27	n, p	5.05 ± 1.79	40.12
Xylocopa latipes	0.11	0.13	1.21	n, p	-	-
Lepidoptera						
Appias libythea	0.06	0.06	0.61	n	-	-
Castalinus rosimon	0.05	0.06	0.56	n	-	-
Catochrysops strato	0.04	0.04	0.40	n	-	-
Eurema blanda	0.06	0.05	0.56	n	-	-
Eurema hecabe	0.05	0.05	0.51	n	-	-
Jamides bochus	0.02	0.03	0.25	n	-	-
Pelopidus mathias	0.07	0.10	0.86	n	-	-
Suastus gremius	0.06	0.11	0.86	n	-	-
Telicota colon	0.05	0.04	0.45	n	-	-
Total visitors	9.32 ± 5.02	10.48 ± 5.55				

note: Abundance- number of individuals/m^2 area/5 min; fl- floral tissue, n- nectar, p- pollen
