# Supplementary material for: Intercropping with Pigeonpea (Cajanus cajan L. Millsp.): An Assessment of Its Influence on the Assemblage of Pollinators and Yield of Neighbouring Non-Leguminous Crops
Source: Life (Basel). 2023 Jan 9;13(1):193. doi: 10.3390/life13010193 (PMC9866136; doi:10.3390/life13010193)
Supplement: Supplementary file 1 [file life-13-00193-s001.zip › Supplementary Table S4.pdf]

Supplementary Table S4. Floral visitors of *Coriandrum sativum* in West Bengal.

Visitors	Abundance		Relative abundance	Floral resources	Umbellete visitation rate	APV
	Distantly fields	Closely fields				
Coleoptera						
Cheilomenes sexmaculata	0.02	0.02	0.41	fl	-	-
Coccinella septumpunctata	0.05	0.04	0.93	fl	-	-
Raphidopalpa foveicollis	0.04	0.05	0.93	fl	-	-
Diptera						
Episyrphus balteatus	0.09	0.10	1.97	n, p	-	-
Eristalinus megacephalus	0.07	0.06	1.35	n, p	-	-
Paragus serratus	0.19	0.17	3.73	p	-	-
Syritta pipiens	0.06	0.07	1.35	n, p	-	-
Hymenoptera						
Amegilla zonata	0.13	0.15	2.90	n, p	-	-
Apis cerana	0.57	0.59	12.02	n, p	8.95 ± 3.33	430.32
Apis dorsata	0.34	0.32	6.84	n, p	8.80 ± 2.67	210.67
Apis florea	0.48	0.51	10.26	n, p	7.10 ± 2.31	291.38
Camponotus compressus	0.08	0.06	1.45	n	-	-
Ceratina binghami	0.32	0.31	6.53	n, p	6.40 ± 1.88	104.48
Chalybion bengalense	0.15	0.16	3.21	n	-	-
Eumenes fraternus	0.11	0.13	2.49	n	-	-
Halictus acrocephalus	0.47	0.48	9.64	n, p	6.95 ± 2.06	200.99
Lasioglossum funebre	0.21	0.20	4.25	n, p	-	-
Tetragonula iridipennis	1.03	1.12	22.28	n, p	2.20 ± 0.83	122.54
Thyreus nitidulus	0.05	0.07	1.24	n	-	-
Lepidoptera						
Appias libythea	0.04	0.05	0.93	n	-	-
Castalinus rosimon	0.02	0.03	0.52	n	-	-
Catochrysops strato	0.03	0.03	0.62	n	-	-
Eurema blanda	0.04	0.05	0.93	n	-	-
Eurema hecabe	0.02	0.03	0.52	n	-	-
Pelopidus mathias	0.04	0.07	1.14	n	-	-
Suastus gremius	0.05	0.08	1.35	n	-	-
Total visitors	4.70 ± 2.58	4.95 ± 2.59				

note: Abundance- number of individuals/m^2 area/5 min; fl- floral tissue, n- nectar, p- pollen
